# Effects of Pelvic Floor Muscle Physiotherapy on Urinary, Bowel, and Sexual Functions in Women with Deep Infiltrating Endometriosis: A Randomized Controlled Trial

**DOI:** 10.3390/medicina60010067

**Published:** 2023-12-29

**Authors:** Simona Del Forno, Laura Cocchi, Alessandro Arena, Valentina Pellizzone, Jacopo Lenzi, Antonio Raffone, Giulia Borghese, Roberto Paradisi, Aly Youssef, Paolo Casadio, Diego Raimondo, Renato Seracchioli

**Affiliations:** 1 Division of Gynaecology and Human Reproduction Physiopathology, IRCCS Azienda Ospedaliero-Universitaria di Bologna, Policlinico di Sant’Orsola, 40138 Bologna, Italy; simona.delfo@gmail.com (S.D.F.); giuliamariaborghese@gmail.com (G.B.);; 2 Department of Medical and Surgical Sciences (DIMEC), University of Bologna, 40126 Bologna, Italy; 3 Department of Biomedical and Neuromotor Sciences, University of Bologna, 40126 Bologna, Italy; 4 Obstetrics and Feto-Maternal Medicine Unit, IRCCS, Sant’Orsola-Malpighi Hospital, University of Bologna, 40126 Bologna, Italy; alyyoussef78@gmail.com

**Keywords:** deep infiltrating endometriosis, 3D 4D transperineal ultrasound, pelvic floor muscles, pelvic floor physiotherapy, dyspareunia

## Abstract

*Background and Objectives*: Endometriosis is a chronic and recurrent disease defined as the presence and proliferation of endometrial glands and stroma outside the uterine cavity. It affects up to 6–10% of women of reproductive age and can be classified into superficial, ovarian, and deep infiltrating endometriosis (DIE). Deep infiltrating endometriosis can be associated with pain symptoms and pelvic floor muscle hypertone. Moreover, it may be responsible of bowel, urinary, and sexual dysfunctions with impairment of women’s quality of life. Few studies have investigated the role of physiotherapy in women with DIE. Here, we aimed first to evaluate the effects of pelvic floor physiotherapy (PFP) on urinary, bowel, and sexual functions. Secondly, we aimed to evaluate the effects of ultrasound visual feedback during PFP on pelvic floor and subjective modifications in the frequency of sexual intercourse. *Materials and Methods*: This randomized controlled trial was conducted between June 2018 and December 2019 at our tertiary center. Nulliparous women with DIE and superficial dyspareunia were enrolled. At first examination, levator hiatal area (LHA) assessed with 3D/4D transperineal ultrasound, pain symptoms, urinary, bowel, and sexual functions were evaluated. Then, women were randomly assigned to no intervention (control group) or treatment with five individual sessions of PFP (experimental group), and after four months women underwent a second examination. Urinary, bowel, and sexual functions were assessed with validated questionnaires at first and second examinations. In particular, the Bristol Female Lower Urinary Tract Symptoms questionnaire was used to evaluate urinary symptoms, the Knowles–Eccersley–Scott–Symptom questionnaire to assess the presence of constipation, and the Female Sexual Function Index to investigate sexual function. Study outcomes were the comparisons among groups in terms of differences in actual changes in median of questionnaire scores between first and second examinations. *Results*: Thirty women (17 in the experimental group and 13 in the control group) completed the study. No significant differences were found between the two groups regarding urinary, bowel, and sexual functions, although women in the experimental group showed a tendency towards an improvement in constipation symptoms. *Conclusion*: In women with DIE, PFP does not appear to affect urinary, bowel, and sexual functions. Therefore, despite the improvement in superficial dyspareunia, chronic pelvic pain, and PFM relaxation with high treatment satisfaction, women should be informed about the unclear impact of PFP on urinary, bowel, and sexual functions. Larger studies are necessary to further investigate the impact of PFP on these functions.

## 1. Introduction

Endometriosis is a chronic inflammatory disease defined as the presence of endometrial-like tissue (glands and stroma) outside the uterine cavity. This condition affects women of reproductive age with an overall prevalence of 5–10% [1]. Endometriosis may involve genital and extragenital locations and it can be classified into superficial, ovarian, and deep infiltrating endometriosis (DIE). Deep infiltrating endometriosis, which is considered the most severe form of the disease, is characterized by the presence of this ectopic tissue that penetrates below the peritoneal surface, involving the pelvic structures and causing adhesions and anatomical distortion [2,3]. Endometriosis may cause pain symptoms, described as chronic pelvic pain, dysmenorrhea, dyspareunia, dysuria, and dyschezia. Moreover, DIE is strongly associated with urinary, sexual, and bowel dysfunctions, with a significant negative impact on women’s health and quality of life [4,5,6,7,8]. Deep infiltrating endometriosis can also be associated with hypertonic or nonrelaxing pelvic floor muscle (PFM) dysfunction, as previously reported [9,10,11]. Nonetheless, PFM coordination and relaxation problems may contribute to bowel, urinary, and sexual dysfunctions [12,13]. 

Transperineal 3D/4D ultrasound (TPU) has demonstrated itself to be an objective, reliable, and noninvasive diagnostic tool for assessing pelvic floor muscle (PFM) morphometry by the measurement of the levator hiatus area (LHA). The LHA is delimited by the puborectalis muscle, symphysis pubis, and inferior pubic ramus, and represents a valuable parameter for the assessment of pelvic floor morphometry both at rest and during dynamic maneuvers, comparable to digital palpation [14,15]. Specifically, the use of 3D/4D-TPU allows for the measurement of dynamic changes in the levator ani muscle hiatal dimensions during pelvic floor contraction and the Valsalva maneuver, allowing for the evaluation of pelvic floor contractility and relaxation capabilities [14,15,16]. Previous studies, which assessed PFM morphometry with TPU, reported smaller LHA in women with DIE in comparison both with healthy women and with women with isolated ovarian endometriosis, supporting the hypothesis of nonrelaxing PFM dysfunction associated with DIE, in particular in women suffering from superficial dyspareunia [6,9,10]. 

Pelvic floor physiotherapy (PFP) stands out as a valid and minimally invasive treatment for addressing PFM dysfunction, including conditions such as pelvic pain syndromes, vestibulodynia, pelvic organ prolapse, urinary and fecal incontinence, obstructed defecation, and sexual dysfunction [17,18,19]. In a recent randomized controlled trial (RCT) [20], PFP proved effective in enhancing pelvic floor muscle relaxation, addressing superficial dyspareunia, and alleviating chronic pelvic pain in women with DIE. However, the impact of PFP on urinary, bowel, and sexual functions remained unexplored in this particular RCT cohort of women [21,22]. Furthermore, as far as our knowledge extends, such an impact has not been assessed in previous studies within the existing literature.

The aim of this study is to report the effect of PFP on urinary, bowel, and sexual functions in women with DIE.

## 2. Materials and Methods

### 2.1. Study Protocol

This study reports the analysis of data on the effect of PFP on urinary, bowel, and sexual functions in a cohort of women with DIE and superficial dyspareunia from a previous RCT conducted between June 2018 and December 2019 at our tertiary center [20]. In brief, the research included women aged 18 to 45 diagnosed with DIE based on clinical and ultrasound assessments (in accordance with the International Deep Endometriosis Analysis Group criteria) [23] and experiencing superficial dyspareunia. Exclusion criteria encompassed a history of or current genital malignancy, pelvic organ prolapse, prior surgery for DIE, ongoing or past pregnancy, congenital or acquired abnormalities of the pelvis or pelvic floor, and the existence of other factors causing chronic pelvic pain. The same criteria are reported in the previously published study [20]. As previously reported [20], 34 women were enrolled in the study. Written informed consent was obtained from all participating women. At first examination data on medical history, age, body mass index (BMI), endometriosis related pain symptoms (i.e., chronic pelvic pain, dysmenorrhea, dyspareunia, dysuria, dyschezia, assessed using a numerical rating scale NRS from 0 to 10) [24] and current hormonal therapy were collected. In particular, dyspareunia (pain during sexual intercourse) was described as superficial, involving pain or discomfort during vaginal entry or at the vaginal introitus, and deep, characterized by pain or discomfort during deeper penetration in the mid or upper vagina. 

Patients were asked to complete the Bristol Female Lower Urinary Tract Symptoms (BFLUTS) questionnaire [25,26] to evaluate urinary symptoms, the Knowles–Eccersley–Scott–Symptom (KESS) questionnaire [27] to evaluate the presence of constipation, and the Female Sexual Function Index (FSFI) [28] to evaluate sexual function (see Supplementary Information). In particular:

- the BFLUTS questionnaire is a validated questionnaire designed to enable the assessment and quantification of the widest range of lower urinary tracts symptoms (LUTS) in women. It is divided into three items to evaluate bladder filling, emptying, and incontinence [25,26] (Supplementary Information S1);
- the KESS questionnaire is a validated questionnaire that includes eleven questions on bowel symptoms, with a total score ranging from 0 (no symptom) to 39 (high symptoms severity). A cut-off score of ≥10 indicates constipated women. This scoring system allows patients to be divided into those suffering from a rectal evacuation disorder (RED), slow-transit constipation (STC), or a combination of the two [27] (Supplementary Information S2);
- the FSFI questionnaire is a validated 19-item questionnaire for assessing key dimensions of sexual function in women. This questionnaire was designed and validated for assessment of female sexual function and quality of life. This questionnaire is made up of a series of items that assess desire, arousal, lubrication, orgasm, sexual satisfaction, and pain related to sexual intercourse [28] (Supplementary Information S3).

Study outcomes were the comparisons among groups in terms of differences in actual changes in median of questionnaires scores (Δ) between first and second examinations.

Each participant underwent 3D/4D-TPU for the assessment of the LHA during rest, maximal contraction, and the maximal Valsalva maneuver. The 3D/4D transperineal ultrasound, conducted by an experienced operator (S.D.F.), preceded the bimanual gynecological examination and transvaginal ultrasound scans to prevent any discomfort. The validated OmniView-VCI technique was employed, as detailed in previous descriptions [14,29]. Using a Voluson E6 ultrasound system (GE Healthcare, Zipf, Austria) with a RAB 8–4-MHz volume transducer, all acquisitions were carried out at an angle of 85° and high rendering quality. The convex volumetric ultrasound transducer was positioned translabially in the midsagittal plane, with the patient in the lithotomy position. The LHA measurements were assessed anonymously offline using dedicated software (4DView 14.4; GE, Zipf, Austria) by one of the investigators, who was blinded to the patients’ clinical data, following the methodology outlined in our previous study [20].

Randomization was performed by assigning each patient randomly to either no intervention (control group) or to receive PFP sessions (experimental group) in a 1:1 ratio. Randomization was computer-generated and concealment of the assignment was ensured by using opaque envelopes which remained sealed until assignment. Therefore, women were randomly assigned to two groups, 17 in the control group and 17 in the experimental group. Four women, all from the control group, were lost to follow-up.

Each patient in the experimental group received information on pelvic floor anatomy and function with the aid of anatomical tables before the start of physiotherapy. A physiotherapist experienced in PFM dysfunction and physiotherapy performed a digital PFM tone evaluation during the first and follow-up examinations after TPU. Pubococcygeus and ischiococcygeus muscle tone was assessed bilaterally at rest, during contraction, and relaxation of the pelvic floor. After the first examination, the women underwent five individual 30 min PFM physiotherapy sessions at weeks 1, 3, 5, 8, and 11. 

During each consecutive session, the women underwent a Thiele massage, a therapeutic intervention involving digital pressure and the subsequent stretching of muscles. This technique aims to induce muscle relaxation, restoring normal pelvic tone and enhancing the coordination of muscle behavior [30,31]. Women in the control group followed standard of care without receiving PFP sessions.

The second assessment was conducted for all participants four months after randomization. During this session, the women were instructed to complete the BFLUTS, KESS, and FSFI questionnaires and rank their endometriosis-related pain symptoms, as the procedures of the initial examination. Additionally, they underwent another 3D/4D-TPU to evaluate the LHA at rest, during the maximum pelvic floor muscle contraction, and during the maximum Valsalva maneuver.

The study protocol received approval from the local institutional Ethics Committee (345/2017/O/Sper), and the trial was registered on ClinicalTrials.gov (IDNCT03572075).

### 2.2. Statistical Analysis

As previously described [20], the calculation of the sample size was based on the effect size of physiotherapy on LHA during maximum Valsalva maneuver that we considered clinically significant. Assuming a baseline LHA20 of 14.5 cm^2^, and a potential increase at the second examination of 0 ± 1 cm^2^ (0%) in the control group and of 1.5 ± 1 cm^2^ (10.3%) in the study group, a total sample of 18 subjects, 9 per group, was required to detect the specified mean difference between the groups with 80% power, using a two-sided 5%-level test [20]. All variables were summarized as median and range (or interquartile range), except a few that did not significantly deviate from normality by means of the Shapiro–Wilk W test, which were summarized as mean ± standard deviation. Actual changes in score (Δ) were preferred over relative changes (Δ%) due to the presence of zero-values on the first examination. Comparisons between groups were performed using the Mann–Whitney U test or Student’s *t*-test, where appropriate, for continuous variables, and Fisher’s exact test or chi-square test, where appropriate, for categorical variables. 

All data were analyzed using Stata software, version 15 (StataCorp. 2017. Stata Statistical Software: Release 15. College Station, TX, USA: StataCorp LLC.). The significance level was set at 5%, and all tests were two-sided. All analyses were intention-to-treat by the originally assigned groups.

## 3. Results

Baseline characteristics of the women studied did not differ significantly between the two groups (Table 1).

No significant differences were found at the second examination compared to the first between the experimental and control group for all questionnaires (Table 2).

Regarding bowel function, 21 (70%) women at first examination reported constipation, of which 11 (65%) in the experimental group and 10 (77%) in the control group, with no significant differences between the groups (*p* = 0.52). In particular, in the experimental group, 10 constipated women (90%) had RED constipation and 1 (10%) had mixed constipation; in the control group, 6 (60%) women had RED constipation, 3 (30%) STC constipation, and 1 (10%) mixed constipation. Comparing the actual change in KESS scores from the first to the second examination among women with RED constipation in the two groups, the decrease in KESS score showed a more pronounced trend in the experimental group, although not reaching statistical significance. Moreover, the improvement in RED constipation symptoms did not reach statistical significance, either overall or by experimental group (Table 3).

## 4. Discussion

### 4.1. Main Findings

PFP does not seem to affect urinary, bowel, and sexual functions in women with DIE, despite the previously reported improvement in superficial dyspareunia, chronic pelvic pain, and PFM relaxation in the same cohort of women [20].

With regard to sexual function, no significant differences were found between the experimental and control groups from first to second examination in the pain domain of FSFI questionnaire. This finding was surprising, especially considering the significant improvement in numeric rating scale (NRS) score of superficial dyspareunia found in women of the experimental group at the second examination [20]. A possible reason may be that we spent time to explain and differentiate superficial dyspareunia (pain occurring in or around the vaginal entrance [32]) from deep dyspareunia (pain in the vagina and pelvis during sexual intercourse [32]) when we asked women to rate pain during sexual intercourse with the NRS, while the pain domain questions of FSFI are not specific for these symptoms. However, to date, there are no dedicated questionnaires to assess and discriminate superficial from deep dyspareunia in women with endometriosis. We believe this topic should be the object of future research. 

Concerning urinary function, no changes after PFP were detected. The women in the study were all women with deep posterior endometriotic lesions not surgically removed, so it is likely that the presence of the lesion itself may be responsible for urinary dysfunctions, which did not improve after physiotherapy. In fact, an increase in the density of nerve fibers and phenomena of perineural and intraneural invasion in DIE lesions have been demonstrated by immunochemistry [33,34,35].

Previous studies investigating urinary function before and after surgery in patients with DIE detected the presence of urinary dysfunction before surgery and did not report improvement after surgery, suggesting a more complex physiopathology of the dysfunction itself [4,8]. Mabrouk et al. [36] evaluated the correlation between the presence of voiding disfunction (according to BFLUTS) and PFM morphometry, using 3D/4D TPU at rest and during dynamic maneuvers in women with posterior DIE. They found that women with voiding dysfunction had levator ani muscle coactivation more frequently than women without voiding dysfunction. Conversely, transperineal measures of LHA at rest and during contraction did not show significant differences between the two groups [36]. In our study, we did not analyze the presence of levator ani muscle coactivation, that may represent a loss of muscle coordination. It might be interesting, in further studies, to evaluate the effects of pelvic floor physiotherapy and visual feedback on levator ani muscle coactivation.

As regards bowel function, chronic constipation with difficult evacuation can be caused by inadequate PFM relaxation and altered coordination [12]. This condition is frequently described as ‘dyssynergic or obstructive defecation’ and is also associated with the inability to coordinate the abdominal, rectoanal, and PFM to evacuate [37]. Manual techniques such as trigger point massage and myofascial release may be useful to correct dyssynergic or obstructive defecation [12]. Moreover, biofeedback therapy is the mainstay for treating the condition, because it permits to “correct the dyssynergia or incoordination of abdominal and pelvic floor muscles during evacuation and improve perception of rectal filling in patients with impaired rectal sensation” [37,38]. In our study, analysis of women with RED constipation after pelvic floor re-education showed a trend towards improvement in both KESS scores and constipation in the experimental group compared to the control group, even if data did not reach significance. We hypothesize that the absence of significance may be probably due to the small number of patients. Moreover, we used Thiele massage, which is not specific for the treatment of constipation. The treatment of women with DIE suffering from RED constipation using dedicated physiotherapy protocols may be an interesting topic for future research. 

Based on these findings and results of other outcomes analysis [20], PFP might be offered to women with DIE in order to improve superficial dyspareunia, chronic pelvic pain, and PFM relaxation with a high grade of treatment satisfaction. Yet, women should be informed about the unclear impact of PFP on urinary, bowel, and sexual functions. We are aware that the small sample size does not allow to draw final conclusion on the effect of PFP on these functions and we cannot exclude a potential impact of PFP with additional PFP sessions. In order to better clarify the effect of PFP on urinary, bowel, and sexual functions, further larger studies are needed.

### 4.2. Strengths and Limitations

The main strength of this study is its originality (it may be the first study in the literature to assess the topic). Additionally, the robust study design, employing a randomized controlled trial (RCT), and the utilization of a noninvasive and reliable technique for evaluating pelvic floor muscles contribute to its credibility. The inclusion of validated questionnaires to assess urinary, bowel, and sexual functions further enhances this study’s methodological strength. Conversely, the main limitation is the relatively small size of the study sample, since it was calculated considering the primary endpoint of the original study [20] and based on the effect size of physiotherapy on LHA during maximum Valsalva maneuver. Therefore, it is likely that enrolling a larger number of patients could yield more informative, reliable, and useful results with regard to urinary, bowel, and sexual functions. Other limitations encompass challenges in standardizing physiotherapy protocols, and notably, the difficulty in identifying a physiotherapy program capable of enhancing all three functions (sexual, bowel, and urinary). The Thiele massage was chosen based on its prior demonstrated effectiveness in treating women with chronic pelvic pain, dyspareunia, and pelvic floor hypertonicity [39,40,41]. Efforts were made to explain the details of the technique better so that it can be easily repeated in future studies. 

## 5. Conclusions

PFP does not appear to affect urinary, bowel and sexual functions in our cohort of women with DIE. Therefore, despite the already reported improvement in superficial dyspareunia, chronic pelvic pain, and PFM relaxation with a high grade of treatment satisfaction, women should be informed about the unclear impact of PFP on urinary, bowel, and sexual functions.

Larger studies are necessary to further investigate the impact of PFP on these functions.

## Figures and Tables

**Table 1:** Baseline characteristics of the women studied (experimental versus control group).

Characteristics	Experimental Group	Control Group	*p*
	(*n* = 17)	(*n* = 13)	
Age (years), mean ± SD	32.5 ± 7.6	32.8 ± 6.7	0.93
BMI (kg/m^2^), median (IQR)	20.9 (19.3–24.7)	19.6 (18.7–22.4)	0.49
Adenomyosis, *n* (%)	11 (65)	7 (54)	0.55
Ovarian cyst, *n* (%)	9 (53)	4 (31)	0.23
Anterior DIE, *n* (%)	0 (0)	0 (0)	1.0
Localization of posterior DIE, *n* (%):			
- Left USL	7 (41)	8 (62)	0.27
- Right USL	5 (29)	3 (23)	1.0
- Rectum	5 (29)	4 (31)	1.0
- RVS	3 (18)	1 (8)	0.61
- Vagina	1 (6)	0 (0)	1.0
Medical Therapy	11 (58)	8 (42)	0.86
Superficial dyspareunia (NRS), median (IQR)	8 (6–10)	8 (7–10)	0.78
Deep dyspareunia (NRS), median (IQR)	7 (4–9)	7 (6–8)	0.72
Dysmenorrhea (NRS), median (IQR)	4 (0–8)	3 (0–6)	0.52
Chronic pelvic pain (NRS), median (IQR)	5 (0–6)	3 (0–5)	0.32
Dysuria (NRS), median (IQR)	0 (0–0)	0 (0–0)	0.66
Dyschezia (NRS), median (IQR)	0 (0–6)	0 (0–4)	0.94
LHA at rest (cm^2^), mean ± SD	10.3 ± 2.1	10.8 ± 2.1	0.53
LHA during contraction (cm^2^), median (IQR)	8.1 (7.6–10.1)	7.7 (7.0–8.4)	0.17
LHA at Valsalva maneuver (cm^2^), mean ± SD	11.4 ± 2.4	12.2 ± 2.2	0.33

Abbreviations: BMI, body mass index; DIE, deep infiltrating endometriosis; USL, utero-sacral ligament; RVS, recto-vaginal septum; NRS, numeric rating scale; IQR, interquartile range; LHA, levator hiatal area.

**Table 2:** Actual change scores from first to second examination (T_0_ to T_1_) in the experimental (physiotherapy) and control (nonphysiotherapy) group; values are median (range) except where otherwise specified.

Scoring	Experimental Group (*n* = 17)			Control Group (*n* = 13)			Δ*_exp_*–Δ*_con_*
	T_0_	T_1_	Δ	T_0_	T_1_	Δ	(*p*-Value)
KESS	14	11	−1	12	12	0	−1
	(2, 23)	(3, 21)	(−9, 7)	(0, 25)	(4, 22)	(−3, 8)	(0.673)
BFLUTS	8	7	−1	9	6	0	−1
	(2, 18)	(1, 21)	(−6, 3)	(1, 17)	(1, 17)	(−8, 12)	(0.916)
Filling	5	4	0	4	2	0	0
	(1, 8)	(1, 8)	(−5, 1)	(1, 7)	(0, 9)	(−7, 5)	(0.576)
Voiding	3	2	0	3	4	0	0
	(0, 8)	(0, 6)	(−5, 3)	(0, 8)	(0, 9)	(−7, 6)	(0.518)
Incontinence	0	0	0	1	2	0	0
	(0, 6)	(0, 7)	(−3, 4)	(0, 6)	(0, 4)	(−3, 2)	(0.740)
FSFI	18.3	20.1	0.0	12.5	13.4	0.9	–0.9
	(1.2, 32.7)	(1.2, 32.5)	(−15.9, 24.4)	(1.2, 26.1)	(1.2, 30.6)	(−15.2, 22.8)	(0.644)
Desire	3.0	3.6	–0.6	2.4	2.4	0.0	–0.6
	(1.2, 5.4)	(1.2, 5.4)	(−1.2, 1.2)	(1.2, 4.8)	(1.2, 4.8)	(−3.0, 3.0)	(0.088)
Arousal	3.3	3.3	0.3	2.1	1.8	0.0	0.3
	(0.0, 5.7)	(0.0, 6.0)	(−5.1, 5.4)	(0.0, 4.8)	(0.0, 4.8)	(−3.3, 4.8)	(0.470)
Lubrification	2.4	3.0	0.0	2.1	1.8	0.0	0.0
	(0.0, 6.0)	(0.0, 6.0)	(−5.4, 5.4)	(0.0, 5.1)	(0.0, 5.4)	(−4.5, 5.1)	(0.637)
Orgasm	4.0	4.4	0.0	1.6	2.0	0.0	0.0
	(0.0, 6.0)	(0.0, 5.6)	(−2.0, 5.6)	(0.0, 5.2)	(0.0, 5.2)	(−4.8, 4.0)	(0.882)
Satisfaction	3.6	4.0	0.0	2.8	3.2	0.0	0.0
	(0.0, 6.0)	(0.0, 6.0)	(−3.6, 5.2)	(0.0, 5.6)	(0.0, 6.0)	(−2.0, 5.2)	(0.672)
Pain	1.6	2.8	1.2	0.0	2.4	0.0	1.2
	(0.0, 3.6)	(0.0, 6.0)	(−3.6, 5.6)	(0.0, 3.6)	(0.0, 5.2)	(−1.6, 5.2)	(0.665)

Abbreviations: KESS, Knowles–Eccersley–Scott–Symptom; BFLUTS, Bristol Female Lower Urinary Tract Symptoms; FSFI, Female Sexual Function Index.

**Table 3:** Actual change in KESS scores from first to second examination (T_0_ to T_1_) in the experimental (physiotherapy) and control (nonphysiotherapy) patients with constipation symptoms (KESS ≥ 10) due to rectal evacuatory disorders (RED) at T_0_ [27]. Improvement in constipation symptoms (KESS ≥ 10) due to rectal evacuatory disorders (RED) [27], overall and by experimental group.

Scoring	Experimental Group (*n* = 9)			Control Group (*n* = 6)			Δ*_exp_*–Δ*_con_*
	T_0_	T_1_	Δ	T_0_	T_1_	Δ	(*p*-Value)
KESS	15	15	–3	14	13.5	–0.5	–2.5
	(14, 23)	(5, 21)	(−9, 7)	(11, 19)	(8, 20)	(−3, 3)	(0.477)
Improvement at T_1_		All	Experimental group		Control group	Exact *p*-value	
		(*n* = 15)	(*n* = 9)		(*n* = 6)		
Yes		6 (40%)	4 (44%)		2 (33%)	>0.99	
No		9 (60%)	5 (56%)		4 (67%)		

Notes: Values are median (range) except where otherwise specified. Improvement is defined as either KESS < 10 or non-RED cause of constipation (slow transit or mixed). Abbreviations: KESS, Knowles–Eccersley–Scott–Symptom.

## Data Availability

The data that support the findings of this study are available from the corresponding author, [D.R.], upon reasonable request.

## Supplementary Materials

The following supporting information can be downloaded at: https://www.mdpi.com/article/10.3390/medicina60010067/s1, Supplementary Information S1: Confidential BFLUTS-SF Questionnaire; Supplementary Information S2: The Knowles–Eccersley–Scott–Symptom (KESS) Questionnaire; Supplementary Information S3: Female Sexual Function Index (FSFI).
