# Supplementary material for: Effects of Pelvic Floor Muscle Physiotherapy on Urinary, Bowel, and Sexual Functions in Women with Deep Infiltrating Endometriosis: A Randomized Controlled Trial
Source: Medicina (Kaunas). 2023 Dec 29;60(1):67. doi: 10.3390/medicina60010067 (PMC10818504; doi:10.3390/medicina60010067)
Supplement: Supplementary file 1 [file medicina-60-00067-s001.zip › medicina-2754392-supplementary.pdf]

Supplement information S1

CONFIDENTIAL BFLUTS-SF QUESTIONNAIRE

F1 During the night, how many times do you have to get up to urinate, on average?

None: 0
1: 1
2: 2
3: 3
4 or more: 4

F2 Do you have to rush to the toilet to urinate?

Never: 0
Occasionally: 1
Sometimes: 2
Most of the time: 3
All of the time: 4

F3 Do you have pain in your bladder?

Never: 0
Occasionally: 1
Sometimes: 2
Most of the time: 3
All of the time: 4

F4 How often do you pass urine during the day?

Every 4 hours or more: 0
Every 3 hours: 1
Every 2 hours: 2
Hourly: 3

BFLUTS-FS: sum scores F1-F4 ……..

V1 Is there a delay before you can start to urinate?

Never: 0
Occasionally: 1
Sometimes: 2
Most of the time: 3
All of the time: 4

V2 Do you have to strain to urinate?

Never: 0
Occasionally: 1
Sometimes: 2
Most of the time: 3
All of the time: 4

V3 Do you stop and start more than once while you urinate?

Never: 0
Occasionally: 1
Sometimes: 2
Most of the time: 3
All of the time: 4

BFLUTS-VS: sum scores V1-V3 ……..

	Total FS	Total VS	Total IS	**TOTAL**
BFLUTS				

I1 Does urine leak before you can get to the toilet?

Never: 0
Occasionally: 1
Sometimes: 2
Most of the time: 3
All of the time: 4

I2 How often do you leak urine?

Never: 0
Once or less per week: 1
2-3 times per week: 2
Once per day: 3
Several times per day:4

I3 Does urine leak when you are physically active, exert yourself, cough, or sneeze?

Never: 0
Occasionally: 1
Sometimes: 2
Most of the time: 3
All of the time:4

I4 Do you ever leak for no obvious reason and without feeling that you want to go?

Never: 0
Occasionally: 1
Sometimes: 2
Most of the time: 3
All of the time: 4

I5 Do you leak urine when you are asleep?

Never: 0
Occasionally: 1
Sometimes: 2
Most of the time: 3
All of the time:4

BFLUTS-IS: sum scores I1-I5 ……..

Supplement information S2

THE KNOWLES-ECCERSLEY-SCOTT-SYMPTOM (KESS) QUESTIONNAIRE	
**1. Duration of constipation**<br>0-18 months = 0<br>18 months to 5 years = 1<br>5-10 years = 2<br>10-20 years = 3<br>>20 years (or all life) = 4	**7. Bloating**<br>Never = 0<br>Perceived by patient only = 1<br>Visible to others = 2<br>Severe causing satiety or nausea = 3<br>Severe with vomiting = 4
**2. Laxative use**<br>None = 0<br>Laxatives prn or for short duration = 1<br>Laxatives regular, long duration = 2<br>Laxatives long duration, ineffective = 3	**8. Enemas / Dictation**<br>None = 0<br>Enemata / suppositories occasionally = 1<br>Enemata/suppositories regular = 2<br>Manual evacuation occasionally = 3<br>Manual evacuation always = 4
**3. Frequency of bowel movement**<br>*(Using current therapy)*<br>1-2 times / 1-2 days = 0<br>2 or less times / week = 1<br>Less than once per week = 2<br>Less than once per 2 weeks = 3	**9. Time taken**<br>*(Minutes in lavatory / attempt)*<br>< 5 minutes = 0<br>5-10 minutes = 1<br>10-30 minutes = 2<br>> 30 minutes = 3
**4. Unsuccessful evacuatory attempts**<br>Never / rarely = 0<br>Occasionally = 1<br>Usually = 2<br>Always = manual evacuation = 3	**10. Difficulty evacuating**<br>*(Causing a painful evacuation effort)*<br>Never = 0<br>Rarely = 1<br>Occasionally = 2<br>Usually 3Always = 4
**5. Feeling incomplete evacuation**<br>Never = 0<br>Rarely = 1<br>Occasionally = 2<br>Usually = 3<br>Always = 4	**11. Stool consistency**<br>*(Without laxatives)*<br>Soft / loose/normal = 0<br>Occasionally hard = 1<br>Always hard = 2<br>Always hard, usually pellet-like = 3
**6. Abdominal pain**<br>Never = 0<br>Rarely = 1<br>Occasionally = 2<br>Usually = 3<br>Always = 4	**KEY**<br>Rarely < 25% of time<br>Occasionally - 25-50% of the time<br>Usually > 50% of the time

Supplement information S3

FEMALE SEXUAL FUNCTION INDEX (FSFI)	
**Q1: Over the past 4 weeks, how often did you feel sexual desire or interest?**	5 = Almost always or always<br>4 = Most times (more than half the time)<br>3 = Sometimes (about half the time)<br>2 = A few times (less than half the time)<br>1 = Almost never or never
**Q2: Over the past 4 weeks, how would you rate your level (degree) of sexual desire or interest?**	5 = Very high<br>4 = High<br>3 = Moderate<br>2 = Low<br>1 = Very low or none at all
**Q3. Over the past 4 weeks, how often did you feel sexually aroused ("turned on") during sexual activity or intercourse?**	0 = No sexual activity<br>5 = Almost always or always<br>4 = Most times (more than half the time)<br>3 = Sometimes (about half the time)<br>2 = A few times (less than half the time)<br>1 = Almost never or never
**Q4. Over the past 4 weeks, how would you rate your level of sexual arousal ("turn on") during sexual activity or intercourse?**	0 = No sexual activity<br>5 = Very high<br>4 = High<br>3 = Moderate<br>2 = Low<br>1 = Very low or none at all
**Q5. Over the past 4 weeks, how confident were you about becoming sexually aroused during sexual activity or intercourse?**	0 = No sexual activity<br>5 = Very high confidence<br>4 = High confidence<br>3 = Moderate confidence<br>2 = Low confidence<br>1 = Very low or no confidence
**Q6. Over the past 4 weeks, how often have you been satisfied with your arousal (excitement) during sexual activity or intercourse?**	0 = No sexual activity<br>5 = Almost always or always<br>4 = Most times (more than half the time)<br>3 = Sometimes (about half the time)<br>2 = A few times (less than half the time)<br>1 = Almost never or never
**Q7: Over the past 4 weeks, how often did you become lubricated ("wet") during sexual activity or intercourse?**	0 = No sexual activity<br>5 = Almost always or always<br>4 = Most times (more than half the time)<br>3 = Sometimes (about half the time)<br>2 = A few times (less than half the time)<br>1 = Almost never or never
**Q8. Over the past 4 weeks, how difficult was it to become lubricated ("wet") during sexual activity or intercourse?**	0 = No sexual activity<br>1 = Extremely difficult or impossible<br>2 = Very difficult<br>3 = Difficult<br>4 = Slightly difficult<br>5 = Not difficult
**Q9: Over the past 4 weeks, how often did you maintain your lubrication ("wetness") until completion of sexual activity or intercourse?**	0 = No sexual activity<br>5 = Almost always or always<br>4 = Most times (more than half the time)<br>3 = Sometimes (about half the time)<br>2 = A few times (less than half the time)<br>1 = Almost never or never
**Q10: Over the past 4 weeks, how difficult was it to maintain your lubrication ("wetness") until completion of sexual activity or intercourse?**	0 = No sexual activity<br>1 = Extremely difficult or impossible<br>2 = Very difficult<br>3 = Difficult<br>4 = Slightly difficult<br>5 = Not difficult
**Q11. Over the past 4 weeks, when you had sexual stimulation or intercourse, how often did you reach orgasm (climax)?**	0 = No sexual activity<br>5 = Almost always or always<br>4 = Most times (more than half the time)<br>3 = Sometimes (about half the time)<br>2 = A few times (less than half the time)<br>1 = Almost never or never
**Q12: Over the past 4 weeks, when you had sexual stimulation or**	0 = No sexual activity

**intercourse, how difficult was it for you to reach orgasm (climax)?**	1 = Extremely difficult or impossible<br>2 = Very difficult<br>3 = Difficult<br>4 = Slightly difficult<br>5 = Not difficult
**Q13: Over the past 4 weeks, how satisfied were you with your ability to reach orgasm (climax) during sexual activity or intercourse?**	0 = No sexual activity<br>5 = Very satisfied<br>4 = Moderately satisfied<br>3 = About equally satisfied and dissatisfied<br>2 = Moderately dissatisfied<br>1 = Very dissatisfied
**Q14: Over the past 4 weeks, how satisfied have you been with the amount of emotional closeness during sexual activity between you and your partner?**	0 = No sexual activity<br>5 = Very satisfied<br>4 = Moderately satisfied<br>3 = About equally satisfied and dissatisfied<br>2 = Moderately dissatisfied<br>1 = Very dissatisfied
**Q15: Over the past 4 weeks, how satisfied have you been with your sexual relationship with your partner?**	5 = Very satisfied<br>4 = Moderately satisfied<br>3 = About equally satisfied and dissatisfied<br>2 = Moderately dissatisfied<br>1 = Very dissatisfied
**Q16: Over the past 4 weeks, how satisfied have you been with your overall sexual life?**	5 = Very satisfied<br>4 = Moderately satisfied<br>3 = About equally satisfied and dissatisfied<br>2 = Moderately dissatisfied<br>1 = Very dissatisfied
**Q17: Over the past 4 weeks, how often did you experience discomfort or pain during vaginal penetration?**	0 = Did not attempt intercourse<br>I = Almost always or always<br>2 = Most times (more than half the time)<br>3 = Sometimes (about half the time)<br>4 = A few times (less than half the time)<br>5 = Almost never or never
**Q18: Over the past 4 weeks, how often did you experience discomfort or pain following vaginal penetration?**	0 = Did not attempt intercourse<br>1 = Almost always or always<br>2 = Most times (more than half the time)<br>3 = Sometimes (about half the time)<br>4 = A few times (less than half the time)<br>5 = Almost never or never
**Q19. Over the past 4 weeks, how would you rate your level (degree) of discomfort or pain during or following vaginal penetration?**	0 = Did not attempt intercourse<br>1 = Very high<br>2 = High<br>3 = Moderate<br>4 = Low<br>5 = Very low or none at all

Domain	Questions	Score Range	Factor	Minimum score	Maximum score
Desire	1,2	1 – 5	0,6	1,2	6,0
Arousal	3,4,5,6	0 – 5	0,3	0	6,0
Lubrication	7,8,9,10	0 – 5	0,3	0	6,0
Orgasm	11,12,13	0 – 5	0,4	0	6,0
Satisfaction	14,15,16	0 (or 1) – 5	0,4	0.8	6,0
Pain	17,18,19	0 – 5	0,4	0	6,0
			Full Scale Score Range	2,0	36,0
